# Swedish social insurance officers' experiences of difficulties in assessing applications for disability pensions – an interview study

**DOI:** 10.1186/1471-2458-7-128

**Published:** 2007-06-27

**Authors:** Berit Ydreborg, Kerstin Ekberg, Kerstin Nilsson

**Affiliations:** 1 Department of Community Medicine and Public Health, Örebro County Council, Örebro Sweden; 2 National Centre for Work and Rehabilitation. Department of Health and Society, Linköping University, Linköping, Sweden; 3 The Sahlgrenska Academy at Göteborg University, Institute of Health and Care Sciences, Göteborg, Sweden; 4 School of Life Sciences, University of Skövde, Skövde, Sweden

## Abstract

**Background:**

In this study the focus is on social insurance officers judging applications for disability pensions. The number of applications for disability pension increased during the late 1990s, which has resulted in an increasing number of disability pensions in Sweden. A more restrictive attitude towards the clients has however evolved, as societal costs have increased and governmental guidelines now focus on reducing costs. As a consequence, the quantitative and qualitative demands on social insurance officers when handling applications for disability pensions may have increased. The aim of this study was therefore to describe the social insurance officers' experiences of assessing applications for disability pensions after the government's introduction of stricter regulations.

**Methods:**

Qualitative methodology was employed and a total of ten social insurance officers representing different experiences and ages were chosen. Open-ended interviews were performed with the ten social insurance officers. Data was analysed with inductive content analysis.

**Results:**

Three themes could be identified as problematic in the social insurance officers' descriptions of dealing with the applications in order to reach a decision on whether the issue qualified applicants for a disability pension or not: 1. Clients are heterogeneous. 2. Ineffective and time consuming waiting for medical certificates impede the decision process. 3. Perspectives on the issue of work capacity differed among different stakeholders. The backgrounds of the clients differ considerably, leading to variation in the quality and content of applications. Social insurance officers had to make rapid decisions within a limited time frame, based on limited information, mainly on the basis of medical certificates that were often insufficient to judge work capacity. The role as coordinating actor with other stakeholders in the welfare system was perceived as frustrating, since different stakeholders have different goals and demands. The social insurance officers experience lack of control over the decision process, as regulations and other stakeholders restrict their work.

**Conclusion:**

A picture emerges of difficulties due to disharmonized systems, stakeholder-bound goals causing some clients to fall between two stools, or leading to unnecessary waiting times, which may limit the clients' ability to take an active part in a constructive process. Increased communication with physicians about how to elaborate the medical certificates might improve the quality of certificates and thereby reduce the clients waiting time.

## Background

The task of the social insurance offices in Sweden is to administer the social insurance system and ensure that people receive the benefits and allowances to which they are entitled [1,2]. In this study we focus on social insurance officers processing applications for disability pensions. In an international comparison of evaluation of disability [3], it was noted that it was difficult to find a common basis for disability pension in fifteen different countries, most of them in Western Europe. There were dissimilarities between the countries, not only regarding the criteria for disability pension, but also in the organisation and control of benefit recipients.

In Sweden applications for disability pensions increased during the late 1990s which has resulted in an increasing number of disability pensions. To decrease sickness rates, clients with long-term sickness absence have to a great extent been granted disability pensions instead of sickness benefit on the initiative of the social insurance officers [4]. This trend is also seen in other European countries. In 2003 approximately 62,000 people in Sweden were granted disability pensions, while five percent of the applications for disability pension were rejected. During the 1990s there was a gradual tightening up on requirements, with more stringent definitions of what constitutes loss of work capacity, based on medical criteria [5]. Reforms have been realized in order to decrease costs related to sickness absence and disability pensions. The social insurance agency as well as the economic security systems is struggling with both structural and financial problems. The agencies in charge of handling complicated cases have to meet the expanding requirements. Many clients do not only fit into one benefit system but have to be handled by more than one agent of the social security system, which demands cooperation and communication between different welfare agents [4,6].

A more restrictive attitude towards the clients has evolved, which has increased the demands on social insurance officers when handling applications for disability pensions. In 1997 stricter regulations came into force and social insurance officers were instructed to coordinate measures between stakeholders, in order to minimize absence from work due to illness and to promote return to work. Psychosocial and labour market aspects were excluded as acceptable reasons for a disability pension. These changes in the social insurance system are expected to influence the social insurance officers' experience of dealing with applications for disability pensions. One consequence found, is that the demands on social insurance officers have increased in recent years and their working conditions have deteriorated [7-9].

The formal basis in the Swedish social insurance system for assessing disability pension and other forms of sickness benefits is solely based on the assumption that work ability is reduced due to medically defined illness. According to Swedish legislation, people whose work capacity is permanently reduced by at least 25 percent as a result of physical or mental illness or impairment are eligible for temporary, full, or partial disability pension [2]. The disability pension is paid on the basis of earnings. The person applies him/herself, or the social insurance office proposes that a long-term sickness benefit should be replaced by a disability pension. An application for disability pension may also be proposed to apply for disability pension by a physician, or by officers at the employment office or social services. Social insurance officers always deal with the applications. In the current study the social insurance officers handled 100–175 cases at the same time. There is a governmental recommendation that clients should return to work or be transferred from long-term sick leave to disability pensions within 12 months, if possible [4]. The social insurance officers summarises all the information of a case to form the basis for a formal decision by the Social Insurance Board. The judgments in the social insurance board will to 90 percent follow the recommendations of the officer reporting the cases [2,10,11]. The decision is based on assessment of the applicant's reduced work capacity due to medical reasons [2] and the judgement is based on a medical certificate. Thus the medical certificates sent to social insurance offices are of great importance. The physician has a major function in completing the extended medical certificate with details of diagnoses and treatment, functional limitations, rehabilitation measures, date of recovery, and, finally, whether or not work capacity is reduced [9]. Earlier studies highlighted that physicians do not receive sufficient knowledge of various working conditions during their training [12-14], and that they find it difficult to keep up with changing social insurance regulations [15]. Social insurance physician has a consultant role on medical matters, and for assessments of the need for additional examinations and rehabilitation. The social insurance physician may refer the client for further assessment by insurance medical specialists such as physiotherapists, qualified social workers, behavioural scientists or physicians with specific competence to improve assessments of work capacity [10].

The traditional role of the social insurance officer has been that of the objective and impersonal civil servant or public service worker, putting the regulations into practice. Changes in social policy have led to an extended role with an increased number of face-to-face encounters with citizens [7]. Hensing et al found three areas where dilemmas in handling clients occur. The first dilemma concerns cooperation with other stakeholders, and occurs because of divergent goals and routines among the various agents. Waiting for medical examinations and medical certificates are examples, as are referrals to the unemployment office, irrespective of the type of benefit dealt with by the social insurance officer. Another dilemma is due to insufficiency in internal routines. Not every situation is covered by legislation, and there is a need for flexibility on the part of the social insurance officers. Finally, client management dilemmas occur, due to difficulties in assessing work capacity. Complicated medical problems, a changing labour market, unemployment and language difficulties, are factors that may complicate the assessment of work capacity.

In a study on clients' meetings with social insurance officers and professionals within the health care sector [16] it was found that women perceived their contact as more supportive than men did. Clients with disability pensions experienced their contact with the social insurance officer as more supportive and empowering than clients without disability pensions. There is a need for more knowledge of how clients in general perceive treatment and contact with professionals working within social insurance and health care. There is also a lack of studies on how social insurance officers perform and practise their work, in particular when the prerequisites are changes in legislation.

The aim of this study was to describe the social insurance officers' experiences of assessing applications for disability pensions after the government's introduction of stricter regulations.

The research questions are:

• How do the social insurance officers experience the basis for the decision- making process according to law and regulations?

• How do the social insurance officers experience facings the clients after the change to stricter regulations?

## Methods

On the basis of descriptions of social welfare, open-ended interviews were judged to be a suitable method for data collection, in order to understand the respondents' perspectives and experiences [17]. The interviews were performed with social insurance officers and conducted using a thematic guide as a basis. The main focus was the process of handling the applications. The open-ended questions allowed the respondents to describe how they experienced their day-to-day work with applications for disability pensions. When using interviews as a method of data collection it is important to remember that interviewing is a way of conversing that is based on and dependent on culturally implicit assumptions about understandings of beliefs, experiences, feelings and intentions [18]. Interaction between the respondent and the interviewer is thus of importance, but in this study it is the content that is in focus, not the interaction.

### Procedure

To reach the social insurance officers, a letter was sent to the heads of four regional social insurance offices, requesting contact with social insurance officers dealing with applications for disability pensions. The interview subjects then voluntarily registered for participation at staff meetings in the four districts. The interviewer selected ten subjects from among the volunteers. They were all contacted by telephone to arrange an interview and informed about the study by the interviewer (BY). They then received a letter presenting the aims of the study and information about their voluntary participation, with a guarantee of confidentiality and a suggestion as to where the interview should take place. All ten were willing to participate in the study. The audio-taped interviews were carried out in Jan-March 2004 at the respondents' workplaces. Each interview lasted about 1–1.5 hours. A secretary experienced in transcribing interviews transcribed the interviews verbatim.

### Participants

The participants in the study were from a region where approximately 100 social insurance officers performed applications for disability pensions. The officers also had to judge other kind of applications as sickness benefits. The educational background for most of the officers was primary or secondary school and in-service training at the social insurance offices. The younger social insurance officers, about 10 percent, had an academic background. The educational background among the participants in this study reflected their category of educational background, but among all staff-members irrespective of their work function, 20 percent had an academic background [19]. Eight women and two men from the region were included, representing different ages, experiences and geographical district. The division between women and men reflected the distribution according to sex among all staff in the social insurance offices in the actual county [19]. Four individuals worked in the main municipality and six in smaller districts. Seven were older than 50 years of age and three were younger, which also corresponded to the general picture of the age of other employees in this organisation [19]. Experience of working in the social insurance office varied between 3 years and 40 years. The participants' different work experience, age and gender were expected to provide variations in their statements about handling applications for disability pensions.

### Analysis

The transcript data were analysed using an inductive content analysis [17]. The material was analysed by the interviewer (BY) and a researcher experienced in analysing qualitative data (KN). They first read and re-read the transcript texts to familiarize themselves with the data. Thereafter units of meaning, i.e. statements and sentences, answering the aim of the study, were noted in the margins. The second step was to group these units in themes according to their content. As the work continued, the content of each theme was expanded or reduced by comparing the themes, and new questions concerning the data arose: questions such as 'What is seen in the groups?' or 'What stands out?' Finally the themes described in the results were reached. Having two separate analysers made it possible to control, at least to some degree, subjectivity and preconceptions in the analysis, and the procedure ensures conformability [20]. The results of agreement in analysing data between the two co-examiners were about 85 %. Points of disagreement were restored through discussion between the authors [21].

### Ethical considerations

This study followed the Humanistic-Social Research Council Ethics Rules [22]. The committee for research ethics at the University of Örebro in Sweden approved the study. Special emphasis was placed on informing the participants about the study, obtaining their consent and treating their statements confidentially. The quotations in the results are used to exemplify the statements of the individual respondents.

## Results

Three themes could be identified in the social insurance officers' descriptions of difficulty in assessing the applications for disability pensions towards a decision on whether the applicant's symptoms or illness qualified them for a disability pension. These are: 1. Clients are heterogeneous. 2. Ineffective and time-consuming waiting for medical certificates impedes the decision process. 3. Perspectives on the issue of work capacity differed among different stakeholders. Each of these themes will be described separately below.

### Clients are Heterogeneous

The typical applicant for a disability pension is an individual who has been on long-term sick leave. This application does not cause the social insurance officer any problems. Neither are re-assessing applicants with a temporary disability pension problematic. Those two kinds of applications represent about 80 percent of all applications. Applications from those who have neuro-psychiatric diagnoses such as ADHD (Attention Deficit Hyperactivity Disorder), Asperger's syndrome or autism are however more difficult to handle, as these assessments are more time-consuming and often demand an extended assessment to obtain clarification of medical status. The client may for example have studied at a special school for some years and the reasons for the applicant's problems in the ordinary school system may not have been made clear. The necessary clarification is obtained in different ways, depending on the individual.

And then you ask yourself: how is it possible that this person has gone through more or less his entire education without anyone really getting to the bottom of what the problems are (R5).

Several social insurance officers also talked about unemployed younger people, less than 30 years of age, as a problematic group of applicants to judge, especially after the regulations had become stricter. These clients may have participated in several activities leading to better health and when they are ready for practical experience at a workplace, the employment office should provide this. In these cases the social insurance officers had to encourage the clients to contact the employment office. Often the social insurance officers experienced problems, as the employment officers tend to be overloaded with ordinary unemployed people and have little time for clients with special needs. The implications are that some clients who are motivated to work risk having to wait passively for employment activities.

They [Employment Officers] are overloaded right now and can't handle any more. But then you've got to look at the way it's organized. I mean, in that case there must be something wrong with it (R5).

Another group of applicants that cause the social insurance officer problems are immigrants and refugees, as some of them suffer from unspecified disorders. This group has also increased in number during the last decade, i.e. during the same period as a tightening-up on public economy in society has occurred. Immigrants and refugees may sometimes 'somatise' their mental symptoms, as interpreted by the social insurance officers. Vague symptoms or health problems are more difficult for the physicians to fit into the diagnosis coding system and it is also more difficult for them to make prognoses about future work capacity. Some social insurance officers also said they felt uncomfortable in encounters with immigrants. One social insurance officer said 'we social insurance officers have to become better at dealing with and understanding other cultures' (R3). Social insurance officers also said they were uncertain about the quality of medical certificates as they do not know if possible difficulties in the client's background, for example torture or experiences of war, have been brought to light in the encounter with the physician.

Then of course there are often psychological problems that haven't been assessed (in the medical certificate). For example they may have been tortured when they perhaps come from a war-torn region (R9).

Social insurance officers also emphasize how difficult it is to judge applications from young, female single parents suffering from stress-related symptoms such as headaches; diffuse neck-shoulder pain and tiredness. There are complexities in these clients' problems, as they often have a low education, several children, and work in low-paid jobs.

I mean, it's a complex picture. It's linked to work, the social situation, and, yes, everything (R4).

The social insurance officers consequently find it understandable that these women with great responsibility and 'strenuous work' develop long-lasting disorders but they have difficulty getting through their applications.

### Ineffective and time-consuming waiting for the medical certificates impedes the decision process

Social insurance officers often have problems in receiving the medical certificate that is required by law as a basis for the disability pension assessment, in reasonable time. It is more difficult for the applicant to be referred to specialists for medical examinations than it is to obtain an examination by a general practitioner at a primary health care centre. This difficulty affects in particular those vulnerable groups described in the previous theme. The social insurance officers say that they often have to remind the physicians to send the certificate, especially specialist certificates.

But this business of getting them to send in a medical certificate can be really, can take a really long time nowadays (R8).

Another problem mentioned by the social insurance officers is that of 'temporary physicians' employed in the primary health care system. The social insurance officers reported a shortage of permanent general practitioners in the informants' region. 'The temporary physicians' do not have the same time to form a deeper relation with the patients compared with the permanent general practitioner, as 'the temporary physician' meets almost every patient only once. This problem of filling medical positions in the primary health care organization causes further delays with the certificates.

And it's the same thing with people being on the sick list for a long time; I think one of the reasons is that the doctors don't have time to get to grips with the problem, or they think 'I'm only here temporarily, I'll just extend it (the sickness certificate). And then the next one comes along and is also only there temporarily (R6).

Even if the medical certificates are received within a reasonable time they are often incomplete and require supplementary assessments or comments if they are to be usable as a basis for the social insurance officer's investigation before a decision is made at the Social Insurance Board.

Sometimes we sort of don't really get answers to what we need. It may be descriptions of different things and functions and so on. But still not a real sort of prognosis, and how long the condition will last, whether it's something that will disappear in a few months or a year, or never. You don't get, they don't take a stand on it, and then it's sort of very difficult to make a decision (R7).

When certificates are incomplete, the social insurance officers need to ask the physicians employed at the Social Insurance Board, who are advisers and consultants on medical matters, for a statement about the need for additional examinations and rehabilitation. Consulting the social insurance physician is time-consuming and may cause further delays if the social insurance physician refers the client for further assessments by insurance medical specialists such as physiotherapists, behavioural scientists or physicians with specific competence to perform assessments of work capacity. In this way the decision-making procedure is prolonged. Social insurance officers sometimes experience these assessments as a waste of time, as the additional information sometimes does not contribute to the basis for judgement of work capacity.

And then maybe you have to wait for six months to a year for the assessment that anyway is going to say no, it's not possible to do anything about it. And that's a big problem. Yes, you've got to wait a long time whatever it is, I think, a really long time (R10).

This occasionally time-consuming waiting for different assessments frustrates the social insurance officers as they feel responsible for the time the client has to wait. This responsibility is reinforced by social insurance officers' experience of feeling over-burdened by a heavy caseload. As long as they has an unsatisfactory assessment to present to the Social Insurance Board, no decision can be made on whether to grant a disability pension or not. The social insurance officer is aware of the problem this may cause the client; for example, not having enough money to pay their bills. One social insurance officer expresses her empathy with the client in the following way:

I mean, I can't just say I don't care about this today if they've got to have money for something; there are dates when bills have to be paid and all that. So this is the pressure you have on you that you've got to get it done by that day; it's their livelihood after all (R1).

The social insurance officers explained that they are responsible for writing a memorandum as a basis for a decision on a disability pension at the Social Insurance Board, even if there is no medical certificate. Presenting an incomplete memorandum and knowing that the application will be rejected by the Social Insurance Board, is expressed as a problem by the social insurance officers, both for the clients as they have to submit a re-application, and for their own job satisfaction.

### Perspectives on the issue of work capacity differed among different stakeholders

The social insurance officers consider that there are difficulties due to the fact that the authorities, such as social insurance offices, employment offices and social services, have different definitions of the concept of work capacity.

Social insurance officers make decisions on work capacity in relation to sickness. Therefore the physicians' certificates are important for the social insurance officers' assessments, but sometimes the physicians avoid judging work capacity.

In the medical certificates they have difficulty formulating what kind of work capacity there is. There's usually a diagnosis and what kind of (medical) problems they have. But how this limits (the individual) in working; they themselves think that's difficult (R9).

Social insurance officers mention that sometimes there may be doubts about which authority is responsible for providing financial support for an individual. In some cases individuals are requested by the employment office to apply for disability pensions, as the employment office regards the client as not healthy enough to be employable. The employment office might also direct clients to apply to the social welfare office for financial support. Here similar problems may occur in the assessment of the individual's health and work ability, i.e. these authorities consider the client too ill to grant unemployment compensation or social welfare. When the applicant comes to the social insurance office he or she may not have been in contact with the health care centre and may be unable to get the necessary medical certificate. When the social insurance officer assesses work capacity in relation to medical status, clients may however be considered healthy enough to be able to work, which eliminates their chance of applying for a disability pension. The fact that social insurance officer and other stakeholders have different interpretations of the concept 'work capacity' is exemplified in the following:

Perhaps we juggle with the applicants, no, but we can say 'then the social welfare office will have to take care of that', and the social welfare office may have thought 'well, but they are rather ill, they haven't got a chance of earning their living'. So of course they're borderline cases everywhere (R10).

## Discussion

The aim of this study was to describe the social insurance officers' experiences of assessing applications for disability pensions during a period with stricter regulations. Interviews with social insurance officers proved to be a suitable method for collecting data for the purpose of the study. All the respondents were motivated to participate, and interested in talking about their experience of the process of reaching a decision on applications for disability pension, which contributed to comprehensive interviews. Even if there is a positive atmosphere during the interview, the interview situation can be affected by interviewees' preconceptions about changes in social insurance regulations and problems this can cause the social insurance officer. Furthermore, the analysis can also be affected by the understanding of the researchers [17]. To minimize these risks, we attempted during the research process to maintain an awareness of our understanding from the field. Generalizing the results from the study is not possible, but the indicated problems that were described in the process may be transferred to similar social insurance units and settings.

The results indicate that applications for individuals from special sub-groups were more complicated to process than those from ordinary clients. As social insurance officers seem to have difficulty assessing some applications in a proper way and in line with the intentions in the primary texts of the law, one must question the extent to which the officers get support and training to interpret changes in laws and legislations. The support given from social insurance physicians seems to be of a more formal character concerning medical certificates and, particularly, the assessment of work capacity.

Söderberg [9] found in her review study of social insurance officers as "gatekeepers" that they sometimes used their decision latitude to prioritize clients who were easier to deal with, e.g. men or non-immigrants, even though this was against the rules. The results in this study do not support Söderberg's findings of prioritizing ordinary groups, but show that social insurance officers find applications from some groups of individuals more difficult to deal with. To manage these problematic applications, social insurance officers may need increased knowledge on how the insurance system should be applied for various clients in order to reduce frustration in the decision-making process and to minimize the lack of equivalence in the decisions. This statement about in-service training is supported by Hensing [7], who points to the fact that social insurance officers have no access to scientific or consensus agreement on which to base their assessments.

Social insurance officers in this study described working with clients with different backgrounds, and that they felt forced to make rapid decisions within a limited time frame, based on limited information mainly from medical certificates. The applications may suffer from insufficient investigations by other stakeholders, which leads to a more difficult and time-consuming decision process for the social insurance officer. This front-line work at the social insurance office could be seen in the light of Lipsky's [23] theory of street-level bureaucrats. He argued that street-level bureaucrats often work with lack of resources, as well as diffuse and ambiguous agency goals, which was essentially confirmed in this study. When social insurance officers process the clients' applications for disability pensions, they represent the policies of the social insurance system and feel forced to live up to rules and legislation [2]. As street-level bureaucrats they are expected to implement public policy when interacting with clients [23]. But Lipsky also argues that the street-level bureaucrats have an opportunity to find their own way of handling the dilemma to be in-between the clients and the policies in society. As mentioned above, Söderberg [9] also found that social insurance officers used their decision latitude to prioritize. Therefore it is interesting to find that the results indicate that social insurance officers virtually uncritically follow the rules and deliver incomplete memorandums to the Social Insurance Board, rather than taking a more offensive attitude to solve the problem of incomplete and delayed medical certificates.

An obvious hindrance in the process of assessing the application was the waiting time, as medical certificates came in late and were often insufficient. Several studies [12-14] emphasize physicians' insufficient knowledge of clients' working conditions. Communication between the two authorities is hampered by the fact that they each have a different basis for evaluation; in medical care the patient's health is the core issue, while the social insurance system focuses on the economic and public service perspectives to be applied to the client's individual needs. The dependence on other stakeholders, in particular physicians, causes social insurance officers to lose control over the process of assessment. In some cases they have a clear opinion on what is best for the client, but they are unable to fulfil this as long as communication with the physician is unsatisfactory or lacking. The social insurance officers may even distrust the physicians' certificates in some cases and be ambivalent regarding these different sets of values. The lack of communication and common goal-setting between the stakeholders causes unnecessary waiting for the client and also makes the decision process more difficult. This 'communication hub' between different stakeholders in getting the client assessed for a disability pension was described by Lipsky [23]. Inadequate communication may partly be due to differences in regulations, values and goals, but may also be a result of differences in perceived position, especially to physicians in the health care system.

It is conceivable that the waiting time frustrates the social insurance officers, as they feel responsible to their clients. Social insurance officers have face-to-face contact with clients but are at the same time obstructed from doing a high quality job; this may cause considerable strain. It was also seen that the long waiting time influenced the decision-making process, as some applications were assessed on an incomplete basis for decision-making. From the client's point of view, such a risk can be perceived as a relief, but at the same time it may be an obstacle for return to work. It may also increase the risk of differences between individual cases in the decisions taken. This interpretation has been described earlier by Hensing [7], who stated that the practice of social insurance officers is of great importance for clients.

The results showed that work capacity, which is a core concept in the decision process, seemed to cause communication problems between different authorities. The problem of communicating work capacity between authorities may have increased when the number of people in need of financial support from society, for example in terms of unemployment benefits, sickness benefits or disability pensions, has increased [5,24]. The social insurance officer's process of decision-making is influenced by ambivalence between empathy for the client on the one hand, and the regulations, which do not accept psychosocial problems or unemployment as a reason for disability pension, on the other. According to Hensing et al [7], changes in social policy have also led to an extended role for social insurance officers, which may contribute to the existing difficulties in interpreting reduced work capacity.

The result implies that there is lack of coordinated actions between authorities. This is a confirmation of Hensing and her colleagues' study [7], where her respondents described the pressure to end long-lasting cases as a "mission impossible", as some were not covered by insurance rules. Social insurance officers raised the question of whether disability pension was a failure that reflected incompetence among actors, or whether it indicated an impossible labour market for individuals with unclear health problems. Lipsky [23] describes it as "a way of dealing with clients in need, without really dealing with them". The social insurance officer is a sort of "gatekeeper" regarding the client's entitlement to disability pension. This "gate-keeping task" in encounters between social insurance officers and clients has not been studied to any great degree in relation to assessing applications for disability pensions. Gard [25] and Klanghed [26] studied similar encounters when rehabilitation professionals worked with people in the rehabilitation process. Reconciliation of differences between various authorities appears to be a core issue in improving welfare processes. Also Friesen [27] and Baril [28] discuss how delays of all types, including ineffective communication among stakeholders, influenced return to work. Friesen [27] indicated how e.g. the insurance system and health service system functioned partly in parallel on one level, while the individuals function in-between on another level.

## Conclusion

The aim of this study was to describe the social insurance officers' experiences of assessing applications for disability pensions after stricter regulations were introduced. Problems described were: the many different clients that could not be considered as uniform, the ineffective and time-consuming delays in communicating with the medical care services and, finally, divergent perspectives on the issue of work capacity among different stakeholders. The different perspectives contributed to the difficulties that occurred in communication between the actors. The role as co-ordinator with other stakeholders in the welfare system was experienced as frustrating, since goals and demands differed between them. The officers worked with clients with manifold backgrounds and had to make rapid decisions within a limited time frame, based on limited information mainly obtained from medical certificates.

A picture emerged of difficulties due to disharmonized systems, stakeholder-bound goals, causing some clients to fall between two stools or leading to unnecessary waiting times, which limited clients' ability to take an active part in a constructive process. To reduce the social insurance officers' stressful working conditions, especially during periods of implementing changes in regulations, it would be of importance with increased management support and training. There was also a need of increased communication with the physicians about how to elaborate the medical certificates as the incomplete certificates often delayed the assessment of applications. The later is not only in favour to the officers but above all to the applicants.

## Competing interests

The author(s) declare that they have no competing interests.

## Authors' contributions

BY: Study design, data collection, data analysis and writing the manuscript.

KE: Study design, participation in writing the manuscript.

KN: Study design, data analysis, writing the manuscript.

All authors read and approved the final manuscript.

## Pre-publication history

The pre-publication history for this paper can be accessed here:


